# Diagnostic value of serum brain-derived neurotrophic factor (BDNF), neuron-specific enolase (NSE), glial fibrillary acidic protein (GFAP), plasma viscosity (PV) as well as fibrinogen (FIB) in acute cerebral infarction patients

**DOI:** 10.5937/jomb0-55111

**Published:** 2025-08-21

**Authors:** Tinghuan Wang, Fei Wang, Xinglu Dong, Xiaofeng Liu, Zhiyu Cui, Yingshuai Shi

**Affiliations:** 1 The First People's Hospital of Jiashan, Department of Neurology, Jiaxing, Zhejiang, China

**Keywords:** serum brain-derived neurotrophic factor (BDNF), neuron-specific enolase (NSE), glial fibrillary acidic protein (GFAP), acute cerebral infarction, butylphthalide and sodium chloride injection, atorvastatin calcium tablets, neurological impairment, serumski neurotrofni moždani faktor (BDNF), neuron-specifična enolaza (NSE), glijalni fibrilarni kiseli protein (GFAP), akutni cerebralni infarkt, injekcija butilftalida i natrijum-hlorida, tablete kalcijum atorvastatina, neurološko oštećenje

## Abstract

**Background:**

To explore the Diagnostic value of serum brain-derived neurotrophic factor (BDNF), neuron-specific enolase (NSE), glial fibrillary acidic protein (GFAP), plasma viscosity (PV) as well as fibrinogen (FIB) in patients receiving butylphthalide and sodium chloride injection in combination with atorvastatin calcium in acute cerebral infarction patients.

**Methods:**

Eighty ACI patients treated at our hospital between January 2022 and January 2024 were included as study participants, followed by divided into the control group (CG) and study group (SG). The CG was given atorvastatin calcium tablets. Based on the CG, the SG received a butylphthalide sodium chloride injection. The clinical efficacy, neurological impairment, daily living ability, hemorheological indicators, neurobiochemical indicators, and occurrence of adverse reactions in the two groups were compared.

**Results:**

Compared to the CG, the SG's total effective clinical effect rate was significantly higher (P<0.05). After therapy, the NIHSS score in the SG showed a significant reduction relative to the CG, and the BI score in the SG was significantly higher relative to the CG (P<0.05). The whole blood high shear viscosity, whole blood low shear viscosity, PV, HCT, and FIB levels in the SG, were significantly reduced relative to the CG (P< 0.05). The improvements of BDNF NSE, and GFAP levels in the SG were significantly superior to the CG (P< 0.05). No significant differences in adverse reactions were observed between the two groups (P>0.05).

**Conclusions:**

The combination of butylphthalide sodium chloride injection and atorvastatin calcium tablets significantly improved clinical outcomes in ACI patients by improving neurological function, daily living ability, cerebral hemodynamics, and neurobiochemical markers. This therapeutic regimen offers a promising approach to ACI management and warrants further clinical promotion. The novel aspect of this study lies in its comprehensive evaluation of both neurological and hemodynamic improvements, highlighting the potential synergistic benefits of this combined therapy.

## Abstract (sr)

**Uvod:**

Cilj je bio da se ispita dijagnostička vrednost serumskog neurotrofnog moždanog faktora (BDNF), neuron-specifične enolaze (NSE), glijalnog fibrilarnog kiselog proteina (GFAP), viskoznosti plazme (PV), kao i fibrinogena (FIB) kod pacijenata koji primaju injekciju butilftalida i natrijum-hlorida u kombinaciji sa kalcijum atorvastatinom kod akutnog cerebralnog infarkta (ACI).

**Metode:**

U studiju je uključeno osamdeset pacijenata sa ACI lečenih u našoj bolnici između januara 2022. i januara 2024., nakon čega su podeljeni u kontrolnu grupu (CG) i grupu za istraživanje (SG). CG je dobijala tablete kalcijum atorvastatina, dok je SG, pored terapije kao u CG, primala i injekciju butilftalida i natrijum-hlorida. Upoređeni su klinička efikasnost, neurološko oštećenje, sposobnost obavljanja dnevnih aktivnosti, hemoreološki pokazatelji, neurobiohemijski pokazatelji i učestalost neželjenih reakcija u obe grupe.

**Rezultati:**

U poređenju sa CG, ukupna stopa kliničke efikasnosti u SG bila je značajno viša (P< 0,05). Nakon terapije, NIHSS skor u SG pokazao je značajno smanjenje u odnosu na CG, dok je BI skor bio značajno viši u odnosu na CG (P< 0,05). Vrednosti viskoznosti pune krvi pri visokom i niskom smicanju, PV, HCT i FIB u IG bile su značajno smanjene u odnosu na CG (P< 0,05). Poboljšanja nivoa BDNF NSE i GFAP u IG bila su značajno izraženija u odnosu na CG (P< 0,05). Nije bilo značajnih razlika u učestalosti neželjenih reakcija između grupa (P> 0,05).

**Zaključak:**

Kombinacija injekcije butilftalida i natrijum-hlorida sa tabletama kalcijum atorvastatina značajno poboljšava kliničke ishode kod pacijenata sa ACI, poboljšavajući neurološku funkciju, sposobnost obavljanja dnevnih aktivnosti, cerebralnu hemodinamiku i neurobiohemijske markere. Ovaj terapijski režim predstavlja obećavajući pristup u lečenju ACI i zahteva dalju kliničku primenu. Novina ove studije leži u sveobuhvatnoj evaluaciji i neuroloških i hemodinamskih poboljšanja, ističući potencijalne sinergijske koristi ove kombinovane terapije.

## Introduction

Acute cerebral infarction (ACI) is a common and serious neurological ischemic cerebrovascular disorder that primarily affects middle-aged and elderly individuals [1]. ACI results from cerebral artery stenosis or occlusion, which causes local ischemia and hypoxia in the brain, leading to tissue softening, necrosis, and, eventually, significant neurological deficits [2]. Clinically, ACI manifests with focal neurological impairments such as hemiplegia, aphasia, vertigo, and ataxia. It is associated with high morbidity, mortality, and disability rates, representing a significant threat to the patient's quality of life and survival [3].

The primary goal of ACI treatment is to restore blood flow to the affected brain regions, improve oxygenation, and promote recovery of neurological function. Common treatment strategies include using antiplatelet drugs, neurotrophic agents, and vasodilators to improve cerebral perfusion and alleviate ischemic damage [4]. Among the various therapeutic agents, butylphthalide, sodium chloride injection, and atorvastatin calcium tablets are frequently used in clinical practice for ACI management [5] [6]. Butylphthalide is a class I brain protectant that enhances cerebral perfusion, alleviating symptoms and reducing ischemic injury [7]. Atorvastatin, widely used for cardiovascular diseases such as coronary heart disease, has proven effects on improving lipid metabolism and offering neuroprotective benefits in ischemic conditions [8].

Despite the individual benefits of these drugs, their combined therapeutic effects in ACI treatment remain inadequately explored. Therefore, this study aims to evaluate the clinical efficacy and therapeutic value of butylphthalide and sodium chloride injection in combination with atorvastatin calcium tablets in treating ACI patients. By focusing on various clinical endpoints, including neurological function, daily living ability, and neurobiochemical markers, this study seeks to provide a deeper understanding of how this combination can enhance ACI treatment outcomes. The experimental approach involves a comprehensive analysis of both clinical and biochemical responses in ACI patients, aiming to determine whether the synergy between these treatments can offer superior benefits compared to conventional therapies.

## Materials and methods

### General data

Eighty patients with ACI who received therapy at our hospital from January 2022 to January 2024 were selected as study subjects. Inclusion criteria: (1) Conformed to the diagnostic criteria of ACI; (2) The diagnosis was validated by brain CT or magnetic resonance examination; (5) The time from onset to admission was <24 h. Exclusion criteria: (1) Other intracranial lesions and intracranial haemorrhage; (2) Combined with multiple organ failure; (5) Had an immune or infectious disease; (4) Combined with malignant tumour; (5) Heart and lung dysfunction; (6) Severe liver and kidney lesions; (7) Allergic to the drugs used in this study. All patients and their families voluntarily signed informed consent.

Demographic and baseline characteristics are summarised in the Results section. All patients were randomly divided into the control group (CG) and study group (SG), each containing 40 cases.

### Methods

Both groups received symptomatic treatment, such as reducing intracranial pressure, hypoglycemia, oxygen inhalation, fluid rehydration, and lowering blood pressure. Aspirin enteric-coated tablets (Sinopyma Group Ouyi Pharmaceutical Co., LTD., Specification: 100 mg) were taken orally, 100 mg/time, once/day. Based on this, the CG adopted atorvastatin calcium tablets (Pfizer Pharmaceutical Co., LTD., Specification: 20 mg) orally, 20 mg/time, once/day.

On the grounds of the CG, the SG was given 100 mL butylphthalide and sodium chloride injection (Shi Pharmaceutical Group Enbipu Pharmaceutical Co., LTD., Specification: 100 mL: butylphthalide 25 mg and sodium chloride 0.9 g) by intravenous infusion, twice daily.

### Observation indicators

(1) Clinical efficacy. The National Institutes of Health Stroke Scale (NIHSS) score decreased by 91%, indicating clinical cure; 46% NIHSS score decreased 90%, indicating obvious effect; 19% NIHSS score decreased 45%, indicating effective; If the NIHSS score decreased by less than 18%, it was considered to be ineffective. Total effective rate = (clinical cure + obvious effect + effective) cases/total cases x100%.

(2) Neurological impairment. The NIHSS was adopted to assess the degree of neurological impairment, scoring 42 points.

(5) Daily living ability. The Barthel index (Bl) was adopted to evaluate daily living ability activity, with a total score of 10 items ranging from 0 to 100.

(4) Hemorheological indicators. The SECCO SA-9000 automatic hemorheological tester measured whole blood high shear viscosity, whole blood low shear viscosity, plasma viscosity (PV), hematocrit (HCT), and fibrinogen (FIB) levels.

(5) Neurobiochemical indicators. 5 mL of fasting venous blood was gathered from patients in the morning and centrifuged at a rate of 5000 r/min to collect serum. Levels of brain-derived neurotrophic factor (BDNF), neuron-specific enolase (NSE), along with glial fibrillary acidic protein (GFAP) were examined by enzyme-linked immunoassay (ELISA).

(6) Both groups were compared to The occurrence of adverse reactions, including fever, myasthenia, diarrhoea, and vomiting.

### Statistical analysis

Data were analysed using SPSS 24.0 statistical software. Measurement data were exhibited as (x±s), and a t-test was adopted for comparison. Count data were displayed as (n, %), and the χ^2^ test was used for comparison. P<0.05 meant statistical significance.

## Results

### Demographic and baseline characteristics

Before therapy, no significant difference was observed between the two groups' demographic characteristics and baseline data (P>0.05). The control group (CG) included 20 men and 20 women aged 42 to 72 (mean: 57.54±5.56 years). The duration of onset was 5 to 14 hours (mean: 8.52±1.42 hours), with infarct sites including the cerebellum (10 cases), basal ganglia (18 cases), and brainstem (12 cases). The study group (SG) included 21 men and 19 women, aged 41-74 years (mean: 57.62±5.65 years), with a duration of onset ranging from 4 to 12 hours (mean: 8.48±1.40 hours). Infarct sites included the cerebellum (9 cases), basal ganglia (19 cases), and brainstem (12 cases). The incidence of hypertension and diabetes was similar between both groups. No significant differences were found in the baseline characteristics between the two groups (P>0.05).

### Clinical efficacy in 2 groups

The total effective clinical response rate was significantly higher in the SG (95.00%) compared to the CG (77.50%) (P<0.05, Table 1), indicating that the combination of butylphthalide sodium chloride injection and atorvastatin calcium tablets resulted in a superior clinical outcome.

**Table 1:** Clinical efficacy in 2 groups.

Groups	Cases	Clinical cure	Obvious effect	Effective	Ineffective	Total effective rate
Control group	40	4	15	14	9	51 (77.50%)
Study group	40	9	16	13	2	58 (95.00%)
χ^2^						5.16
P						0.02

### Neurological impairment in 2 groups

**Figure 1:**
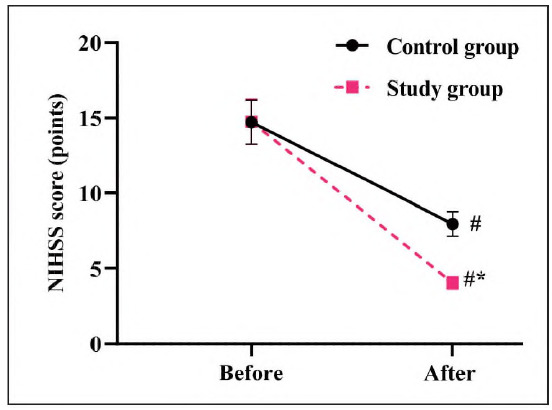
Neurological impairment in 2 groups.

Before treatment, there was no significant difference in NIHSS scores between the two groups (P > 0.05). After therapy, the NIHSS score decreased in both groups. However, the SG exhibited a significantly greater reduction in NIHSS score compared to the CG (P<0.05, Figure 1), suggesting a more effective improvement in neurological function in the SG.

### The activity of daily living ability in 2 groups

**Figure 2:**
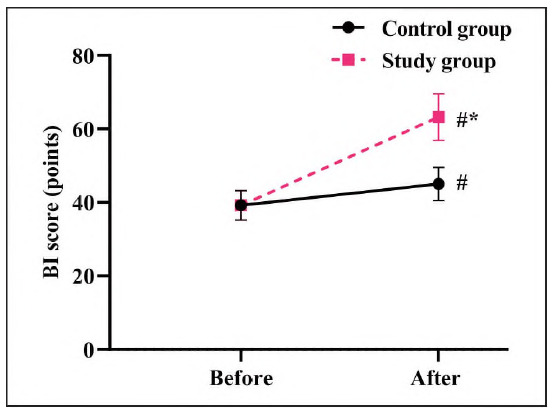
Activity of daily living ability in 2 groups.<br>#P<0.05, in contrast to before therapy, *P<0.05, in comparison to CG.

There was no significant difference in Bl scores between the two groups before treatment (P>0.05). After therapy, the Bl scores increased in both groups, with the SG showing a significantly greater increase in Bl scores compared to the CG (P<0.05, Figure 2), indicating improved functional independence in the SG.

### Hemorheological indicators in 2 groups

**Figure 3:**
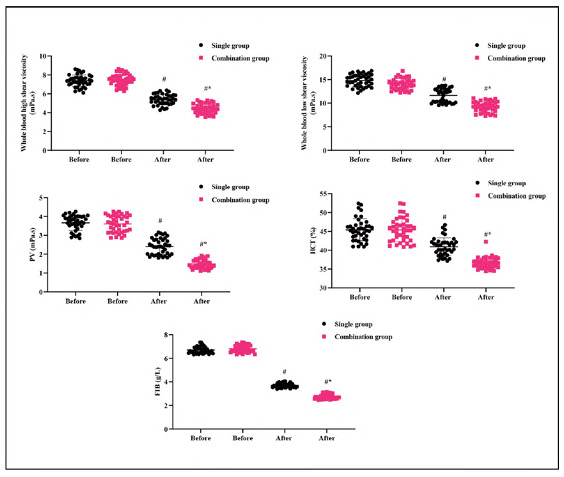
Hemorheological indicators in 2 groups.<br>#P<0.05, in contrast to before therapy, *P<0.05, compared to CG.

Before treatment, there were no significant differences in hemorheological indicators between the two groups (P>0.05). After therapy, the whole blood high shear viscosity, whole blood low shear viscosity, plasma viscosity (PV), hematocrit (HCT), and fibrinogen (FIB) levels decreased in both groups. The SG showed significantly lower levels in all these hemorheological indicators compared to the CG (P<0.05, Figure 3), indicating improved cerebral hemodynamics in the SG.

### Neurobiochemical indicators in 2 groups

**Figure 4:**
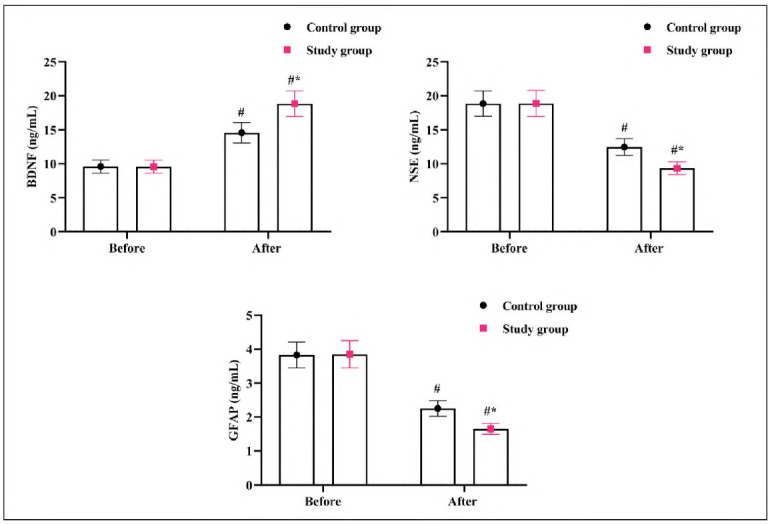
Neurobiochemical indicators in 2 groups.<br>#P<0.05, in contrast to before therapy, *P<0.05, compared to CG.

Before treatment, there were no significant differences in neurobiochemical markers between the two groups (P>0.05). After therapy, BDNF levels increased while NSE and GFAP levels decreased in both groups. The SG showed significantly more significant improvements in these neurobiochemical indicators than the CG (P<0.05, Figure 4).

The neurobiochemical findings provide important insights into the mechanisms underlying the therapeutic effects of the treatment.

BDNF is a critical neurotrophic factor in neuronal survival, synaptic plasticity, and neurogenesis. In this study, elevated BDNF levels in the SG after treatment suggest enhanced neuroprotection and brain tissue repair, which likely contributed to the observed improvement in neurological function. BDNF's role in promoting functional recovery post-ACI is well-documented, and the increase in the SG supports the idea that this combination therapy aids in neurogenesis and synaptic repair.

NSE is a marker of neuronal injury and is released into the bloodstream when neurons are damaged. The reduced levels of NSE in the SG post-treatment reflect less neuronal damage than the CG. This finding indicates that the combination therapy likely mitigates ischemic brain injury, which could be a major factor contributing to the better clinical outcomes observed in the SG.

GFAP is a marker of astrocyte activation and neuroinflammation. A decrease in GFAP levels post-treatment in the SG suggests reduced neuroinflammation, a key component in the progression of ACI. Decreased glial activation in the SG indicates that the therapy may have modulated the inflammatory response, promoting better recovery and preventing further damage. In contrast, the higher GFAP levels in the CG point to ongoing neuroinflammation, impairing neuronal recovery and exacerbating functional deficits.

Together, these biomarker changes suggest that the combination of butylphthalide sodium chloride injection and atorvastatin calcium tablets not only improves cerebral blood flow but also reduces neuronal damage, enhances neuroprotection, and modulates neuroinflammation, all of which are crucial for improving recovery and neurological function in ACI patients.

### Occurrence of adverse reactions in both groups

The incidence of adverse reactions was not significantly different between the two groups (P > 0.05, Table 2). The total incidence rate of adverse reactions was 10.00% in the CG and 15.00% in the SG, suggesting that the combination therapy did not lead to a higher risk of adverse effects than the CG.

**Table 2:** Occurrence of adverse reactions in both groups.

Groups	Cases	Fever	Myasthenia	Diarrhea	Vomiting	Total incidence
Control group	40	1	1	1	1	4 (10.00%)
Study group	40	1	1	2	2	6 (15.00%)
χ^2^						0.46
P						0.50

## Discussion

In recent years, the incidence of ACI has been on the rise with the intensification of the ageing of the population along with the alterations of people's living and working styles. The disease progresses rapidly, and the prognosis is poor, which not only seriously influences the physical and mental health of patients but also brings a tremendous economic burden to the family and society [9]. ACI patients often have severe neurological damage due to cerebral ischemia and hypoxia [10]. Therefore, timely restoration of local blood and oxygen supply in an early stage of the disease and reduced cerebral nerve injury are of positive significance for improving prognosis [11].

Atorvastatin is a hydroxymethylglutaryl coenzyme A reductase, a lipid-lowering drug [12]. Many studies have shown that atorvastatin has pleiotropic effects in addition to lipid regulation, such as anti-oxidative stress, anti-inflammation, inhibition of platelet aggregation, anti-thrombosis, improvement of endothelial function, and stabilisation of plaque, and has certain anti-cerebral ischemia effects [13]. Atorvastatin calcium tablets are a commonly used statin drug in clinical practice, which can relieve the insufficient blood supply to the brain as well as accelerate the recovery of nerve function by reducing the inflammatory response of ACI, inhibiting thrombus formation, promoting cerebrovascular hyperplasia and other ways [14].

Butylphthalide and sodium chloride injection is the first-line drug for treating ACI [15]. This drug is a new class I drug, which can elevate the content of prostaglandin along with reducing the expression of intracellular calcium ions. At the same time, the drug can inhibit the release of glutamate and the formation of tetraenoic acid, stimulate oxidase activity, and play a role in inhibiting inflammatory response [16]. Butylphthalide can enhance blood flow in the cerebral ischemic region, improve microcirculation in the cerebral ischemic region and promote angiogenesis, accelerate cerebral blood flow in the ischemic region, effectively inhibit or block brain injury caused by ischemic cerebrovascular disease, improve cerebral vascular stenosis, reduce cerebral oedema, reduce cerebral infarction area, and minimise brain tissue damage in patients [17]. Meanwhile, it can greatly enhance brain tissue's antioxidant capacity and protect brain nerve function [18].

In our study, the outcomes displayed that relative to the CG, the total effective clinical effect rate in the SG presented elevation, the NIHSS score in the SG presented lower relative to the CG, and the Bl score in the SG presented higher relative to the CG, implying that butylphthalide and sodium chloride injection in combination with atorvastatin calcium tablets was effective in treating AMI, could relieve the degree of neurological impairment as well as restore limb function, thereby improving patients' daily life ability, which was consistent with previous studies [19].

Our study further explored the impact of this combination therapy on key biomarkers. BDNF, a neurotrophic factor crucial for nerve repair, was significantly elevated in the SG compared to the CG [20] [21]. This suggests that the combined treatment promoted neuroprotection and potentially enhanced neuronal plasticity. NSE, a sensitive marker of neuronal damage, was significantly lower in the SG, indieating reduced neuronal Injury [22]. Similarly, GFAF) reflecting astrocyte activation and blood-brain barrier disruption, was also lower In the SG, suggesting Improved Integrity and reduced Inflammation [23] [24]. These changes In blomarkers directly correlate with the observed clinical Improvements In NIHSS and Bl scores. The reduced whole blood high and low shear viscosity, plasma viscosity (PV), hematocrit (HCT), and fibrinogen (FIB) levels In the SG Indicate Improved cerebral hemodynamics, which likely contributed to the enhanced neuroprotectlve effects. These results confirm the plelotroplc effects of atorvastatln and butylphthalIde, demonstrating their ability to modulate neurochemical and hemodynamic factors In ACI. Our findings align with previous studies showing the benefits of atorvastatln and butylphthallde In ACI [25] [26] [27]. However, this study adds value by explicitly examining the combined effect of these two agents and correlating their Impact with changes In multiple blomarkers. The consistent Improvement In clinical outcomes and blomarker levels reinforces the efficacy of this combination therapy.

This study has certain limitations. The sample size was relatively small, which may limit the general- Izablllty of the findings. Additionally, the study duration was limited, and the long-term effects of the combination therapy were not assessed. Future research should Include larger, multicenter trials with longer follow-up periods. Investigating the specific mechanisms through which atorvastatln and butylphthallde Interact and Influence blomarker levels would also be valuable. Furthermore, exploring the potential of these blomarkers as predictors of treatment response and long-term outcomes In ACI patients Is warranted.

## Conclusion

This study demonstrated that combining butylphthalide and sodium chloride Injection with atorvastatln calcium tablets significantly Improved clinical outcomes, Including neurological function and dally living abilities, In patients with acute cerebral Infarction. The Improvements were associated with favourable changes In cerebral hemodynamics and neuroblochemlcal blomarkers, suggesting a comprehensive neuroprotectlve effect. This study provides valuable Insights Into the synergistic effects of these two therapies In ACI, advancing our understanding of effective therapeutic strategies. While not overstating the results, the study highlights the potential of this combination therapy as a promising approach for enhancing ACI treatment.

## Dodatak

### Acknowledgements

We thank all the participants and their families for their cooperation and consent In this study. We also acknowledge the support provided by our Institution's medical and research staff for their dedication and contributions to this project.

### Conflict of interest statement

All the authors declare that they have no conflict of Interest In this work.
